# Comorbidity profiles of psoriasis in Taiwan: A latent class analysis

**DOI:** 10.1371/journal.pone.0192537

**Published:** 2018-02-06

**Authors:** Chen-Yi Wu, Hsiao-Yun Hu, Chung-Pin Li, Yiing-Jeng Chou, Yun-Ting Chang

**Affiliations:** 1 Department of Dermatology, Taipei Veterans General Hospital, Taipei, Taiwan; 2 Institute of Public Health and Department of Public Health, National Yang-Ming University, Taipei, Taiwan; 3 Department of Dermatology, National Yang-Ming University, Taipei, Taiwan; 4 Department of Education and Research, Taipei City Hospital, Taipei, Taiwan; 5 Division of Gastroenterology and Hepatology, Department of Medicine, Taipei Veterans General Hospital, Taipei, Taiwan; 6 National Yang-Ming University School of Medicine, Taipei, Taiwan; Universita degli Studi di Roma Tor Vergata, ITALY

## Abstract

**Background:**

Psoriasis is associated with many comorbidities. An understanding of these comorbidity patterns can help foster better care of patients with psoriasis.

**Objective:**

To identify the heterogeneity of psoriasis comorbidities using latent class analysis (LCA).

**Methods:**

LCA was used to empirically identify psoriasis comorbidity patterns in a nationwide sample of 110,729 incident cases of psoriasis (2002–2012) from the National Health Insurance database in Taiwan.

**Results:**

The mean age of incident psoriasis was 46.1 years. Hypertension (28.8%), dyslipidemia (18.9%), and chronic liver disease/cirrhosis/hepatitis (18.1%) were the top three comorbidities in patients with psoriasis. LCA identified four distinct comorbidity classes among these patients, including 9.9% of patients in the “multi-comorbidity” class, 17.9% in the “metabolic syndrome” class, 11.3% in the “hypertension and chronic obstructive pulmonary disease (COPD)” class, and 60.9% in the “relatively healthy” class. Psoriatic arthritis was evenly distributed among each class. Relative to membership in the “relative healthy” class, an increase of one year of age had a higher probability of membership in the “multi-comorbidity” (odds ratio [OR], 1.25), “metabolic syndrome” (OR, 1.11), or “hypertension and COPD” (OR, 1.34) classes. Relative to membership in the “relative healthy” class, compared to women, men had a higher probability of membership in the “multi-comorbidity” (OR, 1.39), “metabolic syndrome” (OR, 1.77), or “hypertension and COPD” (OR, 1.22) classes.

**Conclusion:**

We observed four distinct classes of psoriasis comorbidities, including the “multi-comorbidity”, “metabolic syndrome”, “hypertension and COPD”, and “relatively healthy” classes, as well as the clustering of liver diseases with metabolic syndrome and clustering of COPD with hypertension.

## Introduction

Psoriasis is a chronic inflammatory disease of the skin with a worldwide prevalence of 0.5–5.5% [1]. The occurrence of psoriasis varies according to age and geographic region, occurring more frequently in countries more distant from the equator [2]. Psoriasis was previously considered to be a disease limited to the skin and joints, but recent evidence suggests that it has far-reaching systemic effects [3]. Psoriasis is associated with various diseases that are characterized by chronic inflammation, including hypertension [4], diabetes mellitus [5], metabolic syndrome [6], stroke [7], coronary artery disease [8], renal disease, liver disease, cancer, and chronic obstructive pulmonary disease (COPD) [3, 9, 10].

Multiple chronic diseases are increasingly placing a greater burden on patients with psoriasis, and these have been investigated individually relative to their association with psoriasis. Such investigations have led to an increasing acknowledgment of the propensity of diseases to occur together, leading to a hypothesis in the “common pathways” implicated in the clustering of diseases [11]. The potential for disease coexistence and clustering should give rise to more attention, as the features of comorbid diseases can be much more complicated than a simple aggregation of individual diseases [12]. Although the need to understand the patterns of disease combinations and their associated complexities is recognized, research on these issues among patients with psoriasis remains limited.

A person-centered approach, such as a latent class analysis (LCA), allows for the examination of how diseases cluster together among individuals, permitting a broader understanding of comorbidity patterns associated with psoriasis. The LCA model has been applied in many domains, including psychology, education, sociology, and behavioral research [13–15]. Latent variable modelling allows investigators to hypothesize the existence of an underlying trait that is not directly measured or observed, but the presence of which can be indirectly inferred by the existence of different observed variables [16]. LCA, which takes multiple diseases into account, enables modelling that is able to holistically scrutinize multiple comorbidities.

This study aimed to identify the distinct profiles of multiple diseases among patients with psoriasis. To accomplish this goal, we applied LCA to a nationwide population of patients with incident psoriasis and analyzed their comorbidity patterns. This person-centered approach took into account the unobserved heterogeneity that is believed to be valuable in understanding the medical burdens faced by patients with psoriasis, and provides new perspectives for physicians to monitor patient need for care and improve the quality of care provided. This knowledge further offer different viewpoints and epidemiological evidences for possible pathophysiological links among psoriasis and comorbidities.

## Materials and methods

### Data sources

The National Health Insurance (NHI) is a mandatory universal health insurance program offering comprehensive medical care coverage to all residents of Taiwan. The NHI program was launched in 1995 to provide health care to all residents, and currently services more than 23 million people, representing approximately 99% of Taiwan’s population. The NHI database contains registration files and original claims data for care reimbursements for all enrollees, making it one of the world’s largest and most complete nation-wide, population-based health care services datasets [17, 18]. In this dataset, the diagnostic codes are formatted according to the *International Classification of Diseases*, *Ninth Revision*, *Clinical Modification (ICD-9-CM)*. Further, the accuracy of major disease diagnoses, such as hypertension, diabetes mellitus, and stroke, has been validated [19, 20]. All information that allows a specific individual patient to be identified is encrypted; thus, the confidentiality of the data abides by the data regulations of the National Health Insurance Administration, Ministry of Health and Welfare, Taiwan. This study was performed in accordance with the Helsinki Declaration and was approved by the National Health Research Institutes and the Institutional Review Board of Taipei Veterans General Hospital (IRB: 2015-02-011CC).

### Nationwide psoriasis cohort

Between 2000 and 2012, we identified a total of 110,729 patients with a first-time diagnosis of psoriasis (ICD-9-CM codes: 696.0 and 696.1). We included cases where the patients had been diagnosed with psoriasis at least three times at outpatient clinics, or were admitted at least once. Baseline data included socioeconomic status (low, medium, and high) and residence location (urban, suburban, and rural) of patients.

### Comorbidities

Information was collected from the NHI dataset. Twelve common chronic diseases were used to determine the latent comorbidity classes. These diseases were identified if they had been diagnosed at least three times previously in the patient or at the same time as the incident psoriasis diagnosis, and included hypertension (ICD-9-CM codes: 401–405), diabetes mellitus (ICD-9-CM codes: 250), dyslipidemia (ICD-9-CM codes: 272), coronary artery disease (ICD-9-CM codes: 410–414), cerebrovascular disease (ICD-9-CM codes: 430–438), connective tissue disease and rheumatoid arthritis (ICD-9-CM codes: 710, 714), renal disease (ICD-9-CM codes: 580–589), chronic liver disease/cirrhosis/hepatitis (ICD-9-CM codes: 571 and 070), COPD (ICD-9-CM codes: 491–496), cancer (ICD-9-CM codes: 140–208), depression and anxiety (ICD-9-CM codes: 296.2, 296.3, 300, and 311), and psoriasis arthritis (ICD-9-CM codes: 696.0).

### Latent class analysis (LCA)

LCA is a statistical method which postulates the homogenous, unobserved subgroups can be identified within a heterogeneous group using a set of observed variables.[16] LCA estimates the response probability for each observed disease according to latent class membership. It also estimates the proportion of individuals that are expected to belong to each latent class. Using information on the comorbidities of patients with psoriasis, LCA was empirically used to identify and estimate the prevalence of diseases patterns. Sex and age were incorporated into the LCA, using logistic regression, as covariates that predict latent class membership.

### Statistical analyses

The demographics of male and female patients were compared using a chi-square test for categorical variables. To identify the optimal LCA model, a sequence of models was examined using two classes, three classes, and so on. A range of indices was used for model selection, including the likelihood ratio G2 statistic, Akaike information criterion, and Bayesian information criterion; model interpretability was also considered. Distinguishability of each class from the others was contemplated on the basis of item-response probabilities; size triviality, such that that no class had a near-zero probability of membership; and the parsimony of the models. Sensitivity analyses were done to perform another LCA that included the existence of comorbidities before and during the 13 years of observation after the incident psoriasis diagnosis. The sensitivity analyses were conducted to examine whether the main findings were robust under different conditions. We used SAS 9.1 (SAS Institute, Cary, NC, USA) to link the data, and Stata 13 (StataCorp, College Station, TX, USA) to perform the LCA, using the Stata LCA plugin [21].

## Results

### Study population characteristics

A total of 110,729 incident cases of psoriasis were identified, including 65,471 (59.1%) men and 45,258 (40.9%) women (Table 1). The mean age of incident psoriasis was 46.1 years (standard deviation [SD], 19.6), with a mean age of 47.6 years (SD, 19.2) for men and 43.9 (SD, 20.0) years for women. Among these patients, 28.8% had hypertension; 14.5% had diabetes mellitus; 18.9% had dyslipidemia; 12.7% had coronary artery disease; 18.1% had chronic liver disease, cirrhosis, and hepatitis; 17% had chronic obstruction pulmonary disease; and 17.1% had depression and anxiety. Compared to psoriatic women, psoriatic men had the most comorbidities. Men had a significantly higher prevalence of hypertension, diabetes mellitus, dyslipidemia, coronary artery disease, cerebrovascular disease, renal diseases, chronic liver disease, cirrhosis, and hepatitis, COPD, and cancer (all, P < 0.001); men also had a lower prevalence of connective tissue disease and rheumatoid arthritis as well as a lower prevalence of depression and anxiety (all, P < 0.001), and psoriatic arthritis (P = 0.008).

**Table 1:** Baseline characteristics of incident psoriatic patients.

	Total		Male		Female		P
	n = 110,729	%	n = 65,471	%	n = 45,258	%	
Age (mean ± SD), year	46.1 ± 19.6		47.6 ± 19.2		43.9 ± 20.0		< 0.001
< 20	9,369	8.5	4,804	7.3	4,565	10.1	< 0.001
20–39	34,606	31.3	18,444	28.2	16,162	35.7	
40–59	37,487	33.9	23,780	36.3	13,707	30.3	
≥ 60	29,267	26.4	18,443	28.2	10,824	23.9	
Socioeconomic status							
Low	58,792	53.1	34,495	52.7	24,301	53.7	<0.001
Medium	31,819	28.7	17,636	26.9	14,183	31.3	
High	20,114	18.2	13,340	20.4	6,774	15	
Urbanity							
Urban	65,004	58.7	37,993	58	27,011	59.7	< 0.001
Suburban	35,740	32.3	21,255	32.5	14,485	32	
Rural	9,985	9	6,223	9.5	3,762	8.3	
Comorbidity							
Hypertension	31,877	28.8	20,251	30.9	11,626	25.7	< 0.001
Diabetes mellitus	16,083	14.5	9,844	15	6,239	13.8	< 0.001
Dyslipidemia	20,921	18.9	12,753	19.5	8,168	18.1	< 0.001
Coronary artery disease	14,018	12.7	8,815	13.5	5,203	11.5	<0.001
Cerebrovascular disease	9,313	8.4	6,026	9.2	3,287	7.3	< 0.001
Connective tissue disease and rheumatoid arthritis	5,197	4.7	2,201	3.4	2,996	6.6	< 0.001
Renal disease	6,063	5.5	3,951	6	2,112	4.7	< 0.001
Chronic liver disease, cirrhosis, and hepatitis	19,982	18.1	13,554	20.7	6,428	14.2	< 0.001
Chronic obstruction pulmonary disease	18,820	17	11,972	18.3	6,848	15.1	< 0.001
Cancer	4,748	4.3	3,103	4.7	1,645	3.6	< 0.001
Depression and anxiety	18,898	17.1	9,489	14.5	9,405	20.8	< 0.001
Psoriatic arthritis	5,955	5.4	3,423	5.2	2,532	5.6	0.008

SD: standard deviation.

### Four classes identified using LCA

**Fig 1:**
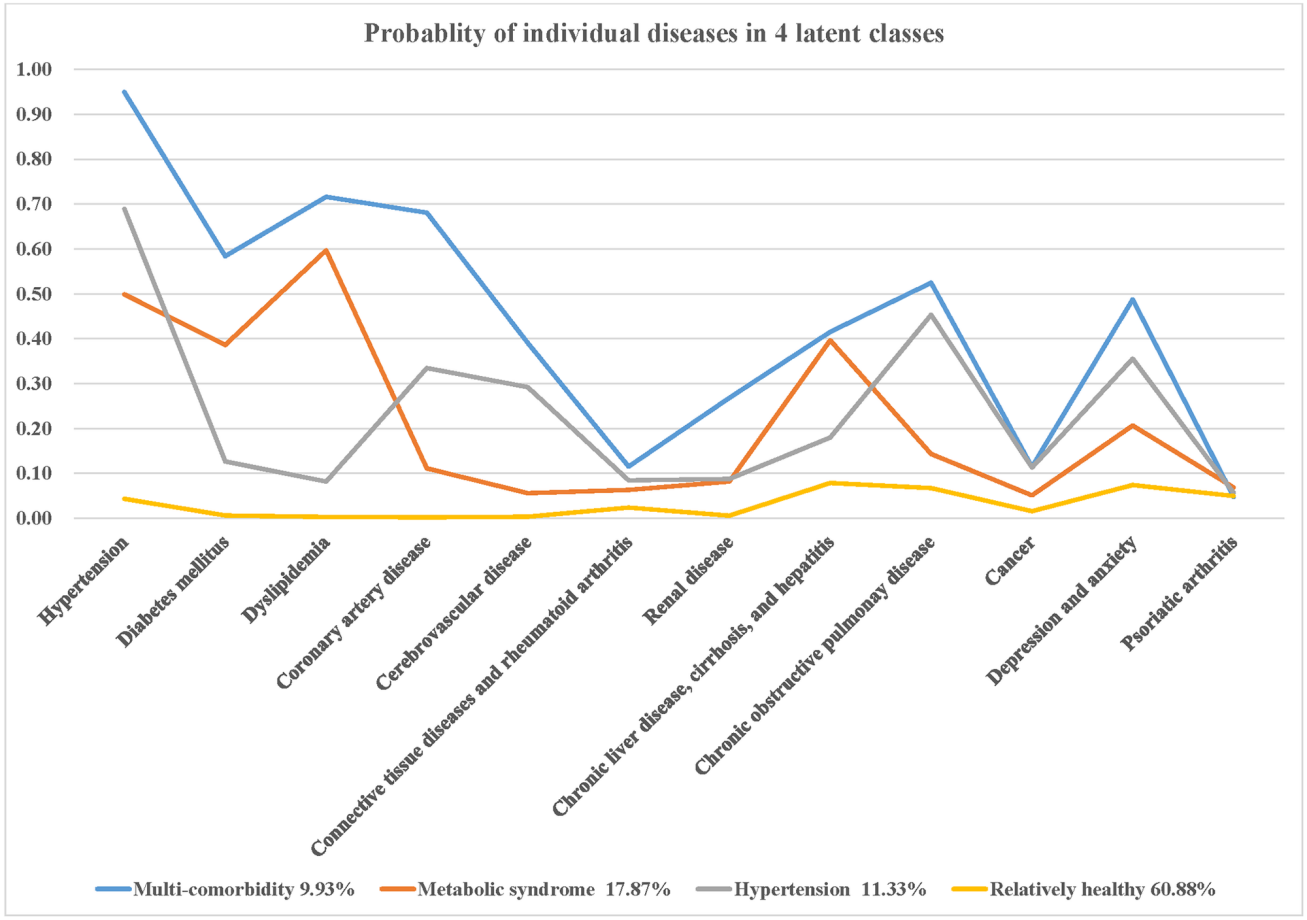
Probabilities of individual disease comorbidities, by latent class. Blue line: class of multi-comorbidity, orange line: class of metabolic syndrome, grey line: class of hypertension and chronic obstructive pulmonary disease (COPD), yellow line: class of relatively healthy.

Having considered the model fit information (Table 2) and model interpretability, the four-class model was selected. The estimated probability of an individual comorbid disease in each class is shown in Fig 1.

**Table 2:** Model fit information for competing latent class models.

No. of classes	Likelihood ratio G^2^	Degree of freedom	AIC	BIC	Entropy
Total					
2	19310	4070	19360	19601	0.81
3	13109	4057	13185	13551	0.71
4	9728	4044	9830	10320	0.73
5	7070	4031	7198	7814	0.68

G^2^: goodness of fit; AIC: Akiake information criterion; BIC: Bayesian information criterion

A label was assigned to each class based on comparisons of the conditional item response probabilities. Four classes were distinguished (Table 3). The first group was characterized by high probabilities of various chronic diseases, including hypertension (95%), diabetes mellitus (58%), dyslipidemia (72%), coronary artery disease (68%), cerebrovascular disease (39%), chronic liver disease and cirrhosis (42%), COPD (52%), and depression and anxiety (49%). Because of the high number of comorbidities, we labeled this group as the “multi-comorbidity” group. The second group was the “metabolic syndrome” group due to the relatively high probability of hypertension (50%), diabetes mellitus (39%), dyslipidemia (60%), as well as chronic liver disease, cirrhosis, and hepatitis (40%). In the third group, “hypertension and COPD,” the individuals had high probabilities of hypertension (69%) and COPD (45%). The last group was the “relatively healthy” group, which was distinguished by a low probability of comorbidities. In total, the four classes of patients with psoriasis, in this study, included 10,962 (9.9%) individuals in the “multi-comorbidity” class, 19,820 (17.9%) in the “metabolic syndrome” class, 12,512 (11.3%) in the “hypertension and COPD” class, and 67,434 (60.9%) in the “relatively healthy” class.

**Table 3:** Item-response probabilities for four class models of total samples: Probability of individual disease in latent class.

	Multi-comorbidity 9.93%	Metabolic syndrome 17.87%	Hypertension and COPD 11.33%	Relatively healthy 60.88%
Hypertension	0.95	0.50	0.69	0.04
Diabetes mellitus	0.58	0.39	0.13	0.01
Dyslipidemia	0.72	0.60	0.08	0.00
Coronary artery disease	0.68	0.11	0.33	0.00
Cerebrovascular disease	0.39	0.06	0.29	0.00
Connective tissue disease and rheumatoid arthritis	0.12	0.06	0.08	0.02
Renal disease	0.27	0.08	0.09	0.01
Chronic liver disease, cirrhosis, and hepatitis	0.42	0.40	0.18	0.08
Chronic obstructive pulmonary disease	0.52	0.14	0.45	0.07
Cancer	0.11	0.05	0.11	0.02
Depression and anxiety	0.49	0.21	0.36	0.07
Psoriatic arthritis	0.05	0.07	0.06	0.05

### Predictors of latent class membership

Table 4 shows the LCA multinomial regression analyses used to examine predictors of latent class membership, with the “relative healthy” class specified as the reference group. This class was used because it was the largest class (60.9% of the participants), representing the majority of the population. Relative to membership in the “relatively healthy” class, an increase of one year of age was more likely to result in the individual being included in the “multi-comorbidity” class (odds ratio [OR], 1.25; 95% confidence interval [CI], 1.25–1.26), “metabolic syndrome” class (OR, 1.11; 95% CI, 1.11–1.11), or “hypertension and COPD” class (OR, 1.34, 95% CI, 1.33–1.35). Relative to membership in the “relatively healthy” class and compared with women, men were more likely to be included in the “multi-comorbidity” class (OR, 1.39; 95% CI, 1.33–1.44), “metabolic syndrome” class (OR, 1.77; 95% CI, 1.68–1.86), or “hypertension and COPD” class (OR, 1.22; 95% CI, 1.13–1.32).

**Table 4:** Predictors of latent class membership.

	Multi-comorbidity OR (95% CI)	Metabolic syndrome OR (95% CI)	Hypertension and COPD OR (95% CI)	Relatively healthy
Age (increased per year)	1.25 (1.25–1.26)	1.11 (1.11–1.11)	1.34 (1.33–1.35)	reference
Gender (male *vs* female)	1.39 (1.33–1.44)	1.77 (1.68–1.86)	1.22 (1.13–1.32)	reference

OR: odds ratio; CI: confidence interval

### Sensitivity analysis

The results of the sensitivity analyses that were used to perform LCAs that included the existence of comorbidities before or during the 13 years of observation after the incident psoriasis diagnosis were robust. The four classes remained distinguished. The four classes of patients with psoriasis included 18,270 (16.5%) in the “multi-comorbidity” class, 18,602 (16.8%) in the “metabolic syndrome” class, 20,263 (18.30%) in the “hypertension and COPD” class, and 53,593 (48.4%) in the “relatively healthy” class.

## Discussion

The findings of this large-scale, nationwide study explored the heterogeneity of comorbidity patterns in patients with psoriasis. We observed four distinct classes of psoriatic comorbidities, including the “multi-comorbidity”, “metabolic syndrome”, “hypertension and COPD”, and “relatively healthy” classes. Fortunately, about 60% of the patients were relatively healthy, with few comorbidities, when they were first diagnosed with psoriasis. In the sensitivity tests that included an observation period of over 13 years, about half (48.4%) of the psoriatic patients remained relatively healthy. Patients with psoriasis and arthritis were almost evenly distributed among the four latent classes. Considerable heterogeneity remained in the remaining psoriatic patients, and these were classified by the LCA into three distinct comorbidity groups. To explore the unobserved heterogeneity by LCA, we observed the clustering of liver diseases with metabolic syndrome, also clustering of COPD with hypertension in psoriatic patients.

In the “multi-comorbidity” class, this class had a high prevalence of hypertension, metabolic syndrome, coronary artery disease, cerebrovascular disease, renal disease, COPD, and psychiatric morbidity. However, cancer and psoriatic arthritis were not particularly clustered into this class. This observation might provide a clue regarding the further evaluation of linkages or pathophysiological mechanisms between these diseases. Also noteworthy is the observation that about half of the patients in the “multi-comorbidity” class presented with depression and anxiety. Further psychiatric evaluations should be considered when caring for patients with psoriasis and multiple comorbidities.

Metabolic syndrome is associated with patients with psoriasis [6, 22]. The “metabolic syndrome” class was the second largest class in our study, and comprised almost one-fifth of the patients with psoriasis. Clustering of liver diseases was also prominent in this class. Interactions linking skin inflammation, non-alcoholic fatty liver disease, and metabolic syndrome have been postulated and studied [23, 24]. All of these diseases share an underlying inflammatory process, and treatment might rely on possible modifiable risk factors, such as sedentary lifestyle, smoking, and alcohol consumption.

Hypertension, having a prevalence of approximately 29% in our study, was observed to be the most common comorbidity in patients with psoriasis. We observed a distinct clustering of “hypertension and COPD”, whereas hypertension alone, without other traditional components of metabolic syndrome, was clustered with COPD. A previous meta-analysis study revealed an increased risk of COPD among patients with psoriasis [25]. Further study might be needed to investigate the epidemiology and possible pathophysiological links among psoriasis, hypertension, and COPD.

Strong psychiatric morbidity has been reported in patients with psoriasis [26, 27]. In one cross-sectional study, about 10–13% of patients with psoriasis also screened positive for major depressive disorder and anxiety disorder [28]. In our study, almost one-fifth of the psoriasis patients presented with depression and anxiety. The majority of these patients clustered into the “multi-comorbidity”, “metabolic syndrome”, and “hypertension and COPD” classes.

Patients with psoriasis and arthritis have been noted to suffer from more severe atherosclerotic disease, metabolic syndrome, and cardiovascular events than patients without arthritis, possibly due to the combination of skin and joint diseases that result in greater systemic inflammation [29, 30]. Psoriatic arthritis patients have heterogeneous clinical presentations. In our study, 5.4% of incident psoriasis patients had presented with psoriatic arthritis. However, they were almost evenly distributed among the four latent classes, including in the “relatively healthy” class. This finding prompts us to examine the possibility of arthritis even in relative healthy patients with psoriasis.

Increased age increases the risks of multiple chronic diseases. A one-year increase in age also resulted in a higher possibility of the individual being classified into the “multi-comorbidity”, “metabolic syndrome”, or “hypertension and COPD” classes. Men with psoriasis also had higher prevalences of most of the comorbidities than did women with psoriasis. In the LCA model, compared to women, men were observed to have higher possibilities of being classified into the “multi-comorbidity”, “metabolic syndrome”, and “hypertension and COPD” classes. This observation reminds physicians to evaluate comorbid conditions, especially when caring for older people or men with psoriasis.

The strength of this study is that our findings are based on a large, contemporary, national cohort of patients with psoriasis. We had a sufficiently large sample size (n = 11,0729) to identify four distinct comorbidity patterns and identify the characteristics associated with each of them. The findings from this person-centered approach, using LCA modelling, provide new perspectives for physicians monitoring comorbidity patterns in patients with psoriasis. As a multi-organ pathology, psoriasis needs a multidisciplinary approach and clinicians should assess it using a holistic view to promptly identify and manage psoriasis comorbidities.

The results of this research should be viewed in light of several limitations. First, the administrative data are subject to possible coding errors and under- or over- coding problems, and this may have affected the prevalence estimates for the different disease patterns. Another limitation is that the LCA model did not examine variations in the longitudinal patterns of the progressing diseases. However, in the sensitivity test that included the existence of comorbidities before or after incident psoriasis, the four classes remained distinguished. Finally, this study was set in Taiwan, the results should be interpreted cautiously as they might not generalize to other races or more heterogeneous populations.

In conclusion, we observed four distinct classes of psoriasis comorbidities, including the “multi-comorbidity”, “metabolic syndrome”, “hypertension and COPD”, and “relatively healthy” classes. As comorbidities are increasingly recognized as being important in the care and management of psoriatic patients, this area requires more attention and better research. The identification of distinct comorbidity patterns offers physicians valuable information that may lead to more appropriate health care, including the exploration of possible pathophysiological associations.
